# Application of Metagenomic Next-Generation Sequencing in the Etiological Diagnosis of Infective Endocarditis During the Perioperative Period of Cardiac Surgery: A Prospective Cohort Study

**DOI:** 10.3389/fcvm.2022.811492

**Published:** 2022-03-08

**Authors:** Xiaodong Zeng, Jinlin Wu, Xin Li, Weiping Xiong, Lili Tang, Xueming Li, Jian Zhuang, Ruoying Yu, Jimei Chen, Xuhua Jian, Liming Lei

**Affiliations:** ^1^Department of Intensive Care Unit of Cardiovascular Surgery, Guangdong Cardiovascular Institute, Guangdong Provincial People's Hospital, Guangdong Academy of Medical Sciences, Guangzhou, China; ^2^Department of Cardiovascular Surgery, Guangdong Cardiovascular Institute, Guangdong Provincial People's Hospital, Guangdong Academy of Medical Sciences, Guangzhou, China; ^3^Guangdong Provincial Key Laboratory of South China Structural Heart Disease, Guangdong Cardiovascular Institute, Guangdong Provincial People's Hospital, Guangdong Academy of Medical Sciences, Guangdong, China; ^4^Dinfectome Inc., Nanjing, China

**Keywords:** infective endocarditis, metagenomic next-generation sequencing, NGS, IE, diagnostic accuracy

## Abstract

**Objective:**

The present study aimed to prospectively evaluate the role of metagenomic next-generation sequencing (mNGS) in the etiological diagnosis of patients with perioperative infective endocarditis (IE).

**Methods:**

From May 1st, 2019 to December 31st, 2020, a total of 99 patients with IE were enrolled in the present study according to the modified Duke criteria, etiological, and pathological results. 11 non-IE patients undergoing heart valve surgery in the same period were selected as the control group. A blood culture test was performed immediately after admission, and the valves harvested operatively were examined by blood culture and mNGS.

**Results:**

In the IE group, there were 29 cases (29.3%) with positive blood culture, 16 cases (16.2%) with positive valve culture, and 85 cases (85.9%) with positive valve mNGS. Compared to culture-based detection, mNGS achieved better performance with a sensitivity, specificity, area under the curve (AUC) of 0.859, 0.727, and 0.793, respectively. The combined approach using culture and mNGS further improved the diagnostic accuracy (sensitivity 89.9%, specificity 72.7%, AUC 0.813). Preoperative white blood cell (*P* = 0.029) and neutrophils (*P* = 0.046) were identified as independent factors affecting the detection rate of mNGS. In the mNGS-positive group, 95 strains of pathogens were found and 10 cases were identified with mixed infection. There were 72 gram-positive bacteria and 14 gram-negative bacteria. mNGS positive group displayed higher species richness than mNGS negative group with enrichment of Streptococcus sanguis, Streptococcus buccalis, and Streptococcus griseus. Proteobacteria and Actinomycetes were enriched in mNGS negative group. Notably, six patients showed disconcordant results between culture and mNGS. Rothia aeria was identified in the blood culture, valve culture, and valve mNGS in one patient. Bartonella Quintana and Coxiella burnetii, which were fastidious intracellular bacteria, were found in two blood and valve culture-negative cases.

**Conclusions:**

mNGS outperformed the conventional culture method and displayed high accuracy in detecting pathogens in IE patients. This study provided support for the use of mNGS in the etiological diagnosis of IE.

## Introduction

Infective endocarditis (IE) refers to the inflammation caused by the direct infection of bacteria, fungi, or other microorganisms, which may dwell in natural or artificial heart valves, endocardial surfaces, or cardiac implanted devices. Valves are the most common affected sites (1). In recent years, the incidence of IE is as high as 7.6–7.8/100,000. The incidence of IE in the elderly population is even up to 37.9/100,000, and the 90-day mortality rate reaches 24.2%, which seriously endangers human health (2). 50% of IE patients need surgical treatment. The size of vegetation, perivalvular infection, and pathogenic diagnosis have a certain impact on the success of surgery (3). The 2015 European Society of Cardiology (ESC) global guidelines for IE pointed out that blood culture was currently the standard method for diagnosis, while etiological diagnosis and drug sensitivity tests can be carried out simultaneously. However, due to the widespread use of antibiotics, some fastidious bacteria and microorganisms are difficult to be identified by traditional methods. The incidence of blood-culture negative endocarditis is as high as 31–35%. The high false-negative rate and the amount of time blood culture take delay timely diagnosis and treatment, which exert a significantly adverse impact on the prognosis (4–6). Therefore, there is an urgent need to improve the accuracy and effectiveness of diagnosis, so as to reduce the mortality of IE patients by early intervention.

Recently, metagenomic next-generation sequencing (mNGS), a powerful platform to sequence and identify nucleic acids from a mixed population of microorganisms, has been applied to pathogen detection in clinical specimens. Compared to the conventional approach, mNGS is known for its high efficiency and accuracy and is expected to become an important clinical diagnostic tool in the future (7). Studies have shown that mNGS achieved sensitivity and specificity of 50.7 and 85.7%, respectively, in the diagnosis of infectious disease pathogens, especially in the detection of viruses, fungi, and anaerobic bacteria (8). Andreas Oberbach et al. studied the valve-related intramural and intracellular bacterial diversity in IE patients and found that polymicrobial infections, pathogen diversity, and intracellular persistence are related to the high mortality of IE patients, while mNGS provides evidence for anti-infection treatment and can improve clinical prognosis (9). Currently, there are few studies on mNGS-based detection of heart valve pathogens. According to a small cohort study (*N* = 11 cases) in 2020, pathogens were detected in microdissected valvular tissue of blood-culture negative IE patients using mNGS, which provides an important basis for the utility of mNGS in IE diagnosis (10). Another study compared several methods, revealing a high sensitivity of 97.6% and specificity of 85.7% for mNGS (5).

Thus, it is believed that a larger cohort is essential to confirm the clinical value of mNGS-based pathogen detection and provide comprehensive information on the distribution of microbiota in IE patients. This study prospectively analyzed the diagnostic value, influencing factors, and microbiota distribution of mNGS in heart valve tissue, yielding solid evidence for the clinical application of mNGS in IE patients.

## Materials and Methods

### Patients and Study Design

A total of 2,352 patients with cardiac valve diseases undergoing valvular surgery in our center during May 1st, 2019, and December 31st, 2020 were enrolled in this prospective study. Ninety-nine patients with IE and 11 patients without IE were finally included (Supplementary Figure 1). The criteria for the diagnosis of IE were based on the modified Duke criteria including etiological results, and pathological results. Inclusion criteria were as follows: 1. patients with infective endocarditis requiring surgical treatment; 2. age ≥ 18 years; 3. written consent for mNGS examination obtained. Exclusion criteria included: 1. patients with severe infection and/or multiple organ dysfunction before operation; 2. Died within 48 h after operation; 3. Patients who did not agree to sign informed consent. The control group was non-IE patients receiving heart valve surgery. This study was approved by the Institutional Review Board (IRB) of Guangdong Provincial People's Hospital. The number is KY-Z-2020-610-02. All patients signed the informed consent.

After admission, the patient's age, height, weight, hypertension, history of cardiac surgery, preoperative usage of antibiotic, white blood cell counts, myocardial markers, inflammatory indicators, liver function, kidney function, blood culture, cardiac color Doppler ultrasound, cardiopulmonary bypass time, aortic clamp time, ICU stay, hospital stay, and surgical outcomes were recorded.

### Blood Culture and Valve Culture

Blood culture and retention refer to the ESC Guidelines for the management of effective endocarditis in 2015 (4). All included patients underwent blood culture examination after admission. Each patient received 3 successive sets of blood culture (including aerobic bottles and anaerobic bottles). Each culture bottle with 10 ml of blood was sent to the clinical microbiology room for culture within 30 min. Valve tissue was collected during the operation, and stored in a sterile culture bottle, and sent to the microorganism room for culture within 30 min. And the valve tissues were also sent for mNGS testing at the same time.

### mNGS Processing

Samples of valves were collected from patients according to standard procedures. DNA was extracted using the QIAamp DNeasy Blood & Tissue Kit (Qiagen). Purified DNA was qualified by Nanodrop 2,000 (Thermo Fisher Scientific) and quantified by Qubit 2.0 using the dsDNA HS Assay Kit (Life Technologies) according to the manufacturer's recommendations. DNA libraries were prepared using the KAPA Hyper Prep kit (KAPA Biosystems) according to the manufacturer's protocols and sequenced on Illumina NextSeq 550Dx (Illumina). High-quality sequencing data were generated by removing low-quality reads, adapter contamination, duplicated, and short (length <36 bp) reads as previously described (11). The human host sequence was identified by mapping to the human reference genome (hs37d5) using bowtie2 (12) software. Reads that could not be mapped to the human genome were retained and aligned with the microorganism genome database for pathogens identification using k-mer-based algorithm with Kraken2 (13, 14). Kraken2 is an ultrafast and highly accurate program for assigning taxonomic labels to metagenomic DNA sequences, which has been used in many mNGS studies (15–17). Kraken2 compares sequence reads with comprehensive metagenome databases, which is different from *de novo* assembly-based methods that do not use any reference databases (17).

Repeated DNA extraction and sequencing with blank tubes filled with UltraPure Dnase/Rnase-free distilled water were used as no-template control (NTC) as previously described (18). The mNGS was considered positive if: (1) For *Mycobacterium, Nocardia*, and *Legionella pneumophila*, a species detected by mNGS had a species-specific read number ≥1; (2) For bacteria (excluding *Mycobacterium, Nocardia*, and *Legionella pneumophila*), fungi, virus, and parasites, a species detected by mNGS had at least 3 non-overlapping reads; (3) Pathogens detected in the negative = NTC were excluded if the detected reads <10-fold than that in the NTC. The QC information of the NGS sequencing was shown in Supplementary Table 1. The median quality score(Q30) was 92.29, indicating the reliability of the mNGS sequencing.

### Statistical Analysis

SPSS 23.0 and R software (3.6.1) was used for data analysis and visualization. Student's t or rank-sum test (Mann-Whitney U) was used to compare the continuous variables, and the categorical variables were analyzed using the Chi-square test. A receiver operating characteristic (ROC) curve was used to evaluate the comprehensive detection efficiency of different detection methods. Sensitivity, specificity, accuracy, negative predictive value, positive predictive value, etc was calculated. *P* < 0.05 indicates that the difference is statistically significant.

All valid sequences of all samples were annotated and classified using Kraken2. The species abundance level of the sample was estimated using Bracken Bayesian method. Alpha diversity was evaluated for each patient using R software. Kruskal Wallis test or one-way ANOVA was used to analyze the difference among the species in the samples between groups, and the species with significant differences were selected.

## Results

### Clinical Characteristic

A total of 110 patients were included in this study, including 99 patients with IE and 11 patients without IE according to the modified DUKE criteria (19) (Table 1; Supplementary Figure 1). Less than 80% of IE patients (78) were males, with an average age of 47 ± 15.7 years and an average weight of 59 ± 11.2 kg. There were 6 males (54.5%) in the non-IE group, with an average age of 52 ± 9.2 years and an average weight of 55 ± 10.2 kg. In the IE group, there were 29 (29.3%) blood culture-positive cases, 16 (16.2%) valve culture-positive cases, and 85 (85.9%) valve mNGS-positive cases. 14 cases were found to be positive in both valve culture and valve mNGS detection. In the non-IE group, there were no positive cases in blood culture or valve culture, but 3 positive cases in valve mNGS (27.3%). In the IE group, there were 3 deaths, 1 case of infectious multiple organ dysfunction, 1 case of severe low cardiac output, and 1 case was discharged automatically due to pulmonary infection and septic shock.

**Table 1:** Clinical characteristics of 110 patients with valvular heart disease.

**Characteristic**	**IE patients (*N* = 99)**	**Non-IE patients (*N* = 11)**	***P*-value**
Male	78 (78.8%)	6 (54.5%)	0.073
Mean age	47 ± 15.7 years old	52 ± 9.2 years old	0.431
Weight	59 ± 11.2 kg	55 ± 10.2 kg	0.218
Left ventricular end-diastolic diameter (mm)	56 ± 8.3	53 ± 4.0	0.132
Left ventricular ejection fraction (%)	61 ± 8.7	58 ± 13.5	0.611
Antibiotics used before admission	43 (43.4%)	2 (18.2%)	0.194
Previous cardiac surgery	9 (9.1%)	0	0.594
Blood culture positive	29 (29.3%)	0	0.035
Valve culture positive	16 (16.2%)	0	0.361
Valve NGS positive	85 (85.9%)	3 (27.3%)	<0.001
Cardiopulmonary bypass time (min)	167 ± 73.7	193 ± 59.3	0.053
Aortic cross-clamp time (min)	111 ± 49.9	131 ± 42.6	0.049
ICU length of stay (d)	4 ± 3.0	4 ± 3.2	0.459
Hospital length of stay (d)	40 ± 12.5	28 ± 13.2	0.007
Death	3 (3%)	0	1.0

### Detection Ability of Different Diagnostic Methods

Next, we evaluated the detection performance of four methods, including blood culture, valve culture, valve mNGS, and combined method of mNGS and culture. As shown in Table 2, valve mNGS displayed a high sensitivity of 85.9% with a specificity of 72.7% compared to blood/valve culture (sensitivity:29.3%/16.2%, specificity: 100%/100%). The combined method further increased the sensitivity to 89.9% with a specificity of 72.7%. The specificity and sensitivity were comprehensively evaluated by the ROC curve (Supplementary Figure 2). It was found that the area under curves (AUC) of mNGS and combined detection in detecting valvular vegetation were 0.793 and 0.813, respectively, which were significantly higher than that of blood/valve culture (0.646/0.581). Univariate analysis was performed to identify the relative factors that may impact mNGS detection (Table 3). The results showed that the potential factors were preoperative leukocyte (*P* = 0.029) and neutrophil value (*P* = 0.046).

**Table 2:** Diagnostic ability of different test modalities for patients with IE.

**Test modality**	**Sensitivity**	**Specificity**	**Positive predict value**	**Negative predict value**	**ROC** **AUC**	**ROC** ***P***
Blood culture	29.3% (0.21–0.39)	100% (0.72–1.00)	100% (0.88–1.00)	13.6% (0.07–0.23)	0.646 (0.601–0.691)	0.112
Valve culture	16.2% (0.10–0.25)	100% (0.72–1.00)	100% (0.79–1.00)	11.7% (0.06–0.20)	0.581 (0.544–0.617)	0.381
Valve NGS	85.9% (0.77–0.92)	72.7% (0.39–0.94)	96.6% (0.90–0.99)	36.4% (0.17–0.59)	0.793 (0.651–0.935)	0.001
Combined detection	89.9% (0.82–0.95)	72.7% (0.39–0.94)	96.7% (0.91–0.99)	44.4% (0.22–0.69)	0.813 (0.672–0.954)	0.001

**Table 3:** Univariate analysis of baseline clinical features that associated with NGS detection ability.

**Clinical features**	**NGS + (*N* = 85)**	**NGS-(*N* = 14)**	**Statistics**	***P*-value**
Hypertension	10	3	0.984	0.321
CAD	10	4	2.796	0.094
Cerebrovascular disease	15	3	0.116	0.734
Previous cardiac surgery	6	3	3.003	0.083
Antibiotics used before admission	37	6	0.002	0.962
AR (cm2)	6.0 (0–12.2)	2.7 (0–9.6)	469.000	0.201
MR (cm2)	8.0 (4.4–13.2)	7.1 (2.7–10.3)	511.500	0.402
LVED (mm)	57.0 (50.0–63.5)	55.0 (51.7–60.2)	561.500	0.736
LVEF (%)	64.0 (60.0–67.0)	66.0 (43.0–69.5)	574.500	0.837
PCT (ng/mL)	0.20 (0.06–0.87)	0.10 (0.01–0.78)	505.000	0.364
CRP (mg/L)	26.40 (8.39–63.06)	13.15 (0.72–50.07)	436.000	0.110
ESR (mm/h)	31.0 (11.0–54.7)	31.0 (11.0–54.7)	558.500	0.764
WBC (10^9^/L)	7.97 (6.67–10.92)	6.42 (5.07–8.54)	377.000	0.029
Neutrophil (10^9^/L)	5.68 (3.89–8.70)	4.01 (3.23–5.97)	396.500	0.046
Lymphocyte (10^9^/L)	1.44 (1.03–1.90)	1.26 (1.09–1.85)	521.500	0.460
Uric acid (umol/L)	372.8 (282.3–451.0)	398.7 (350.6–471.8)	481.000	0.277
BNP (pg/mL)	2,433.0 (546.7–4655.5)	2,758.5 (128.8–4709.7)	554.500	0.684
Troponin (pg/mL)	24.7 (11.7–96.2)	20.6 (8.8–59.9)	539.500	0.577
CK (U/L)	40.0 (29.0–71.0)	43.5 (32.7–153.5)	480.000	0.247
CKMB (U/L)	10.0 (10.0–12.0)	10.0 (5.09–13.6)	588.500	0.946

*CAD, coronary artery disease; AR, aortic regurgitation; MR, mitral regurgitation; LVED, center ventricular end-diastolic diameter; LVEF, center ventricular ejection fraction; PCT, procalcitonin; CRP, C-Reactive protein; ESR, erythrocyte sedimentation rate; WBC, white blood cell; BNP, B-type natriuretic peptide; CK, creatine kinase; CKMB, creatine kinase-MB*.

### Microbiota Analysis of IE Patients

**Figure 1:**
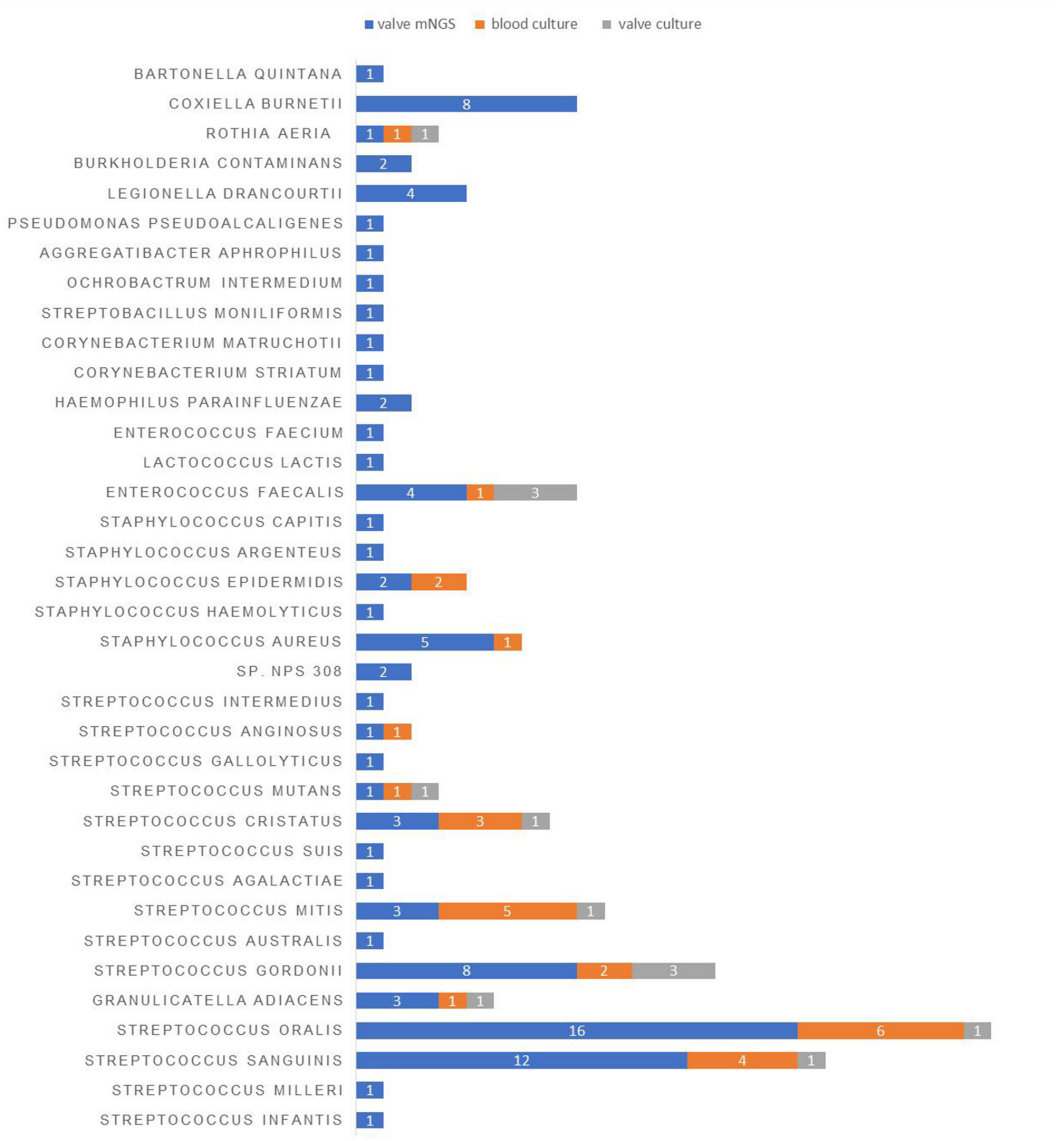
The distribution of pathogens detected in the blood and valve samples of IE patients using different detection methods.

Next, we looked into the distribution of microbiota in the valve tissue of 99 IE patients using mNGS. A total of 95 pathogens strains and 10 cases of mixed infection were found. There were 72 gram-positive bacteria, 14 gram-negative bacteria, and 9 atypical pathogens including Coxiella burnetii and Bartonella Quintana (Figure 1). The commonly-identified bacteria were 16 strains of Streptococcus oral, 12 strains of Streptococcus sanguinis, 8 strains of Streptococcus gordonii, 8 strains of Coxiella burnetii, 5 strains of Staphylococcus aureus and 4 strains of Legionella drancourtii, and 3 strains of Granulicatella adiacens, Streptococcus mitis, Streptococcus cristatus and Enterococcus faecalis (Table 4).

**Table 4:** Summary of representative cases identified in valve NGS.

**Classification**	**Case**	**Blood culture**	**Valve culture**	**Valve NGS (Reads)**	**Antibiotics used**
False positive cases	1	Negative	Negative	Staphylococcus hominis (8) Streptococcus oralis (3)	None
	2	Negative	Negative	Legionella drancourtii (10)	
	3	Negative	Negative	Enterococcus faecium (277)	
Disconcordant cases	4	Abiotrophia defective	Negative	Streptococcus mitis (71)	Imipenem cilastatin sodium combined with vancomycin
	5	Streptococcus mitis streptococcus oralis	Negative	Streptococcus gordonii (113)	
	6	Streptococcus anginosus	Streptococcus gordonii	Streptococcus gordonii (979)	
	7	Streptococcus mitis	Negative	Streptococcus oralis (357)	
	8	Streptococcus mitis	Negative	Streptococcus oralis (1,132,57)	
	9	Streptococus constelltus	Streptococcus gordonii	Streptococcus gordonii (1,196,702)	
Cases with uncommon pathogens	10	Rothia aeria	Rothia aeria	Rothia aeria (20,125)	Imipenem cilastatin sodium combined with vancomycin
	11	Negative	Negative	Bartonella Quintana (287,213)	
	12–19	Negative	Negative	Coxiella burnetii (118–1,559,253)	Doxycycline, imipenem cilastatin sodium, vancomycin

**Figure 2:**
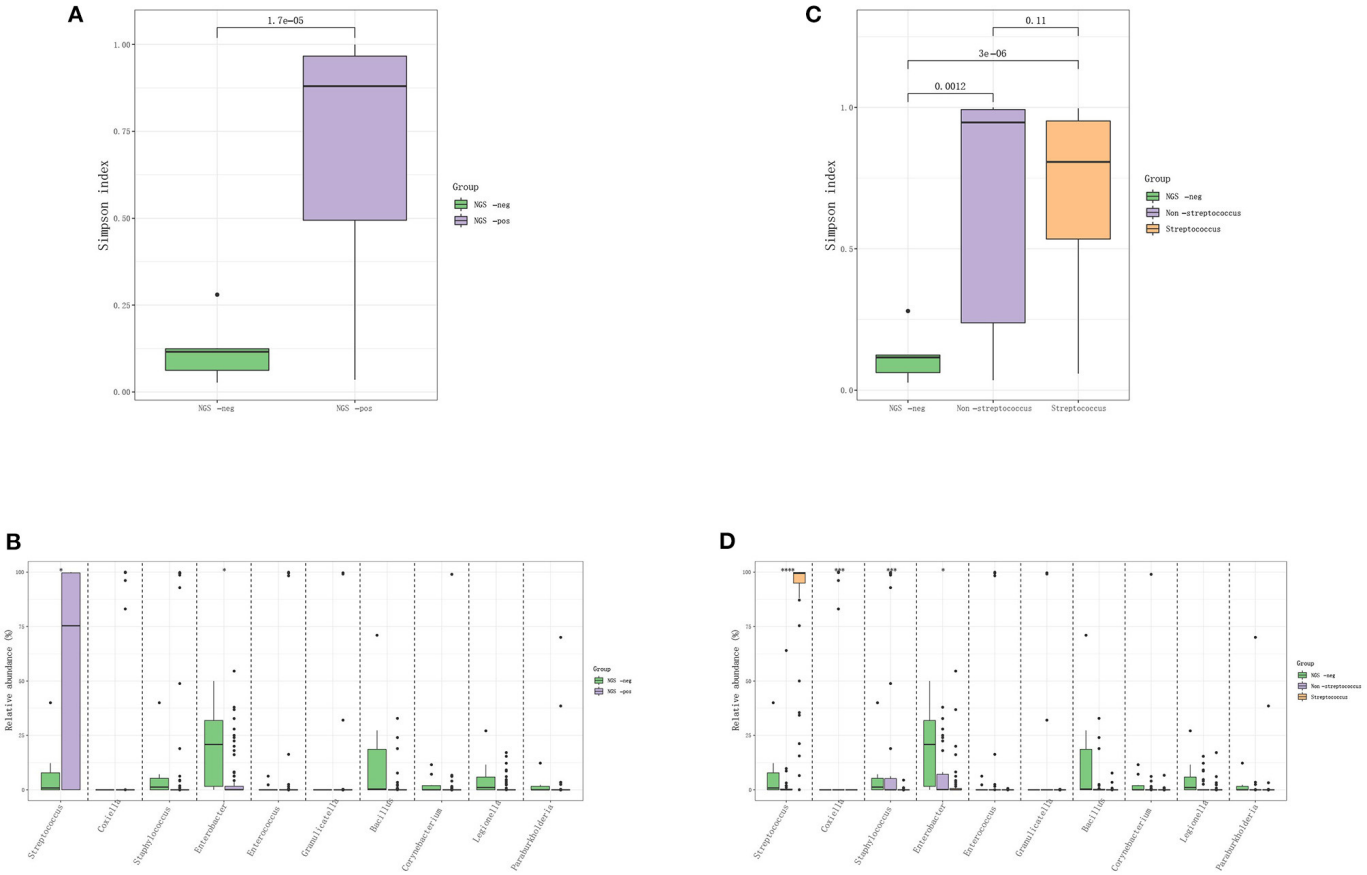
The difference in the distribution of pathogens between mNGS negative and mNGS positive IE patients. **(A,B)** Comparison of Simpson index and species-level difference between mNGS negative group and mNGS positive group; **(C,D)** comparison of ACE index and species level difference among mNGS negative group, mNGS positive non-streptococcal infection group, and mNGS positive streptococcal infection group.

The IE patients were divided into the mNGS-negative group (*N* = 14) and mNGS-positive group (*N* = 85), and the diversity of their microbiota was analyzed to construct a landscape of valve microbial composition. As shown in Figure 2, mNGS-positive and mNGS-negative patients had a different composition of valve microbiota, and the mNGS-positive group had higher species richness than the mNGS-negative group. The dominant species in valves from the mNGS-positive group were Firmicutes, such as Streptococcus sanguis, Streptococcus buccalis, and Streptococcus griseus, while the dominant species in the mNGS-negative group were Proteobacteria and Actinomycetes, such as Enterobacter cloacae. The abundance of Streptococcus in the mNGS-positive group was significantly higher than that in the mNGS-negative group, while the abundance of Enterobacter was significantly higher in the mNGS-negative group. We further divided mNGS-positive patients into mNGS-positive non-streptococcal infection group (*N* = 35) and mNGS-positive streptococcal infection group (*N* = 50) for analysis. The alpha diversity index (Simpson index and Ace index) of mNGS negative group was the lowest compared to the two mNGS-positive groups. The species composition of mNGS negative group was similar to that of mNGS positive non-streptococcal infection group, and the dominant species at the phylum level were all Proteobacteria. Compared to the mNGS-positive streptococcal infected group, the abundance of Staphylococcus and Coxiella burgdorferi in the non-streptococcal infected group was significantly higher.

### Representative Cases in Valve mNGS Detection

In mNGS testing, 3 out of 11 non-IE patients were detected with pathogens, including Staphylococcus hominins, Streptococcus oralis, Legionella drancourtii, and Enterococcus faecium (Table 4). Meanwhile, the mNGS results of 6 IE patients were inconsistent with the traditional culture (Table 4). The blood culture of Case 4 showed infections with hypoxic deficient bacteria, while the mNGS was Streptococcus mitis. The blood/valve culture and mNGS results of Case 5, 6, 7, 8, and 9 were all Streptococcus, but the specific species were different. Notably, cases with uncommon pathogens were identified in IE patients (Table 4). Case 10 was identified with Rothia aeria in blood culture, valve culture, and mNGS testing. Rothia aeria is a rare gram-positive bacterium. Patients with Rothia aeria infection may be complicated with severe embolic events and need to be operated on as soon as possible, especially when the anti-infection effect is poor (20, 21). According to the results of mNGS, which was faster than the culture result, imipenem cilastatin sodium combined with vancomycin were administrated in this patient. The course of treatment is 36 days and the condition of the patient was improving. In nine culture-negative cases, mNGS showed that 1 case was infected with Bartonella Quintana and 8 cases were infected with Coxiella burnetii. Imipenem cilastatin sodium combined with vancomycin were administrated according to the mNGS result. Doxycycline was administrated in addition to the treatment of imipenem and vancomycin in the eight cases identified with Coxiella burnetii. All patients experienced clinical remission and were discharged from the hospital.

## Discussion

Patients with severe infective endocarditis are often accompanied by heart failure and embolic events, leading to increased mortality. Early surgery and effective anti-infective treatment is the key to improving the outcomes (3, 22). However, there is still an unmet need for a faster and more accurate method of IE diagnosis. To solve this problem, this study compared and analyzed culture-based and mNGS-based pathogenic diagnoses in IE patients. The mNGS displayed several advantages compared to the conventional culture-based methods. First, mNGS is much more efficient than the culture method which normally takes about 1 week or more. Most mNGS results were reported within 48 h, similar to other studies (23, 24). Second, the mNGS method outperformed culture methods and reached a high ROC of 0.793 in the analyzed cohort. In addition, mNGS technology is less affected by the use of preoperative antibiotics. Even the detection of killed bacterial debris can provide a basis for the choice of the clinical antibiotic, which also reflects the advantages of high-throughput and unbiased testing (7, 25). Lastly, cases with mixed infection were identified using mNGS but not in blood/valve culture, indicating mNGS may have better performance in distinguishing mixed infection. Therefore, mNGS-based detection is a promising method for the rapid and accurate diagnosis of IE etiology. On the other hand, during blood culture, a drug sensitivity test can be carried out at the time to guide anti-infection treatment (19). Furthermore, the combined method of mNGS and culture may be preferred due to its best performance with a sensitivity, specificity, and accuracy of 89.9%, 72.7%, and 81.3%, respectively, in this study.

Another study reported that the positive rate of blood culture was 42% (950/2267) (26), which was higher than ours. It was speculated that this study only included patients who required surgery, and 43.4% of these patients had preoperative antibiotic treatment. In addition, it was difficult to culture fastidious bacteria and rare pathogens through traditional technology.

Interestingly, preoperative white blood cell counts and neutrophil counts were identified as two potential factors that affect the detection of mNGS. It was reported that the mortality increased by 2.3 times in patients with infective endocarditis if the white blood cell count increased by 5^*^10^9^/L (27). Carlo Tascini et al. studied the relationship between inflammatory indicators and mortality in IE infected with Staphylococcus aureus. They found that PCT, white blood cell, and C-reactive protein were important factors associated with mortality (28). This showed that the increased leukocytes were closely related to the mortality of IE patients, especially IE with Staphylococcus aureus infection. Therefore, patients with increased leukocytes may be regarded as eligible recipients for mNGS testing. Other inflammatory indicators, such as erythrocyte sedimentation rate and C-reactive protein, did not show statistical differences in this study, but it has been confirmed in previous studies that the increased levels of these two inflammatory indicators were closely related to the poor outcomes in infective endocarditis (29).

In recent years, infective endocarditis caused by Staphylococcus and Streptococcus has increased up to 80% of total cases and accounted for 25–30% of IE cases with Staphylococcus aureus infection (30). In this study, Streptococcus and Staphylococcus were also the main pathogens found in the mNGS-positive IE group. The common bacteria in gram-positive bacteria were Streptococcus (56/95, 58.9%), Staphylococcus (10/95, 10.5%) and Enterococcus (4/95, 4.2%). In developed countries, staphylococcus is the most common bacteria of IE due to intravenous administration, hemodialysis, and other factors, which is different from developing countries (1). The positive detection rate of HACEK group infections in blood cultures, which are fastidious gram-negative organisms with high requirements for microbial laboratory culture conditions, is low even in patients with high suspicion of IE (29). In this study, 2 cases of Haemophilus parainfluenzae and 1 case of Haemophilus mumophilus were detected by mNGS, which provides a rapid and accurate basis for clinical treatment.

Of note, this study found that 3 out of 11 non-IE patients are false positive and the result of 6 cases was inconsistent between mNGS and traditional culture. Several factors might contribute to the difference: (1) The blood samples for culture were sampled during admission while the valve tissue subjected to mNGS was collected during operation. Antibiotics had been used for a while in some patients before operation and may have killed most of the bacteria. Interestingly, the culture and mNGS results of cases 5, 6, 7, 8, and 9 were all Streptococcus, but the specific populations were different, suggesting that antibiotics may have different efficacies in treating different populations of Streptococcus; (2) The bacteria detected in valve tissue by mNGS may be bacterial debris (5). Yet, we lacked the micro-transcription analysis of valve tissue; (3) The valve and sampling conditions may also affect the test results (31).

The incidence of blood-culture negative endocarditis (BCNE) is about 20–35%, even as high as 69% in developing countries, leading to increased embolic events, aggravated valve regurgitation, and heart failure. It has always been a challenge for clinical diagnosis and treatment (6, 32, 33). mNGS provides a solution to this problem. In this study, 8 cases of Coxiella burnetii and 1 case of Bartonella quintana were found in the cases with negative blood and valve culture. Coxiella burnetii endocarditis is an important cause of BCNE, which can be acute, subacute, or chronic. The pathogen is a highly fastidious intracellular bacterium, which often leads to treatment delay due to difficult diagnosis. The valvular disease found by transthoracic color Doppler ultrasound is a potentially important risk factor (34–36). Here Coxiella burnetii was efficiently detected by mNGS, and the sequence reads fluctuated from 118 to 1559,253. All 8 patients with Coxiella burnetii in mNGS testing changed the antibiotics regimen according to the results of mNGS and had a good prognosis. It is reported that mNGS technology has greater advantages in patients with negative blood culture, which suggests that mNGS technology can be a better surrogate for pathogen detection in highly suspected IE patients with negative blood culture (37, 38). More studies are warranted to reveal the effect of mNGS result on the prognosis of the IE patients.

The limitations of this study were the small non-IE cohort size and the mNGS results were not verified by other methods such as PCR, which would have a certain impact on the data interpretation. Further study with a larger sample size was warranted to validate our observation.

In conclusion, our data supported mNGS as a preferred method in the etiological diagnosis of IE during the perioperative period of cardiac surgery, especially for patients with negative blood cultures and high suspicion of IE.

## Data Availability Statement

The datasets presented in this study can be found in online repositories. The names of the repository/repositories and accession number(s) can be found at: https://www.ncbi.nlm.nih.gov/bioproject/PRJNA784716/.

## Ethics Statement

The studies involving human participants were reviewed and approved by Guangdong Provincial People's Hospital. The patients/participants provided their written informed consent to participate in this study.

## Author Contributions

JC, XJ, and LL conceived and supervised the study. XZ, JW, XiL, WX, LT, XuL, and JZ performed the experiments and acquired clinical information. XZ, JW, XiL, WX, LT, XuL, JZ, and RY performed data analysis and wrote the manuscript with the help of all of the authors. All authors participated in data interpretation, provided critical revision, and consent the approve of the manuscript.

## Funding

This study was supported by Medical Scientific Research Foundation of Guangdong Province, China (No. A2021486); National Natural Science Funds of China (Grant No. 81900285); Science and Technology Program of Guangzhou, China (Grant No. 202002030317); Guangdong Basic and Applied Basic Research Foundation (Grant No. 2020A1515010242); Guangdong Provincial Clinical Research Center for Cardiovascular Disease (2020B1111170011); National Key R&D Program of China (No. 2018YFC1002600); Guangdong peak project (No. DFJH2019); Science and Technology Planning Project of Guangdong Province, China (No. 2019B020230003); Guangdong peak project (No. DFJH201802).

## Conflict of Interest

RY is the employee of Nanjing Geneseeq Technology Inc. RY is employed by Dinfectome Inc, China. The remaining authors declare that the research was conducted in the absence of any commercial or financial relationships that could be construed as a potential conflict of interest.

## Publisher's Note

All claims expressed in this article are solely those of the authors and do not necessarily represent those of their affiliated organizations, or those of the publisher, the editors and the reviewers. Any product that may be evaluated in this article, or claim that may be made by its manufacturer, is not guaranteed or endorsed by the publisher.
