# Supplementary material for: Application of Metagenomic Next-Generation Sequencing in the Etiological Diagnosis of Infective Endocarditis During the Perioperative Period of Cardiac Surgery: A Prospective Cohort Study
Source: Front Cardiovasc Med. 2022 Mar 8;9:811492. doi: 10.3389/fcvm.2022.811492 (PMC8965566; doi:10.3389/fcvm.2022.811492)

**Figure S1. Patient flowchart.** IE: infective endocarditis. mNGS: metagenomic next-generation sequencing.


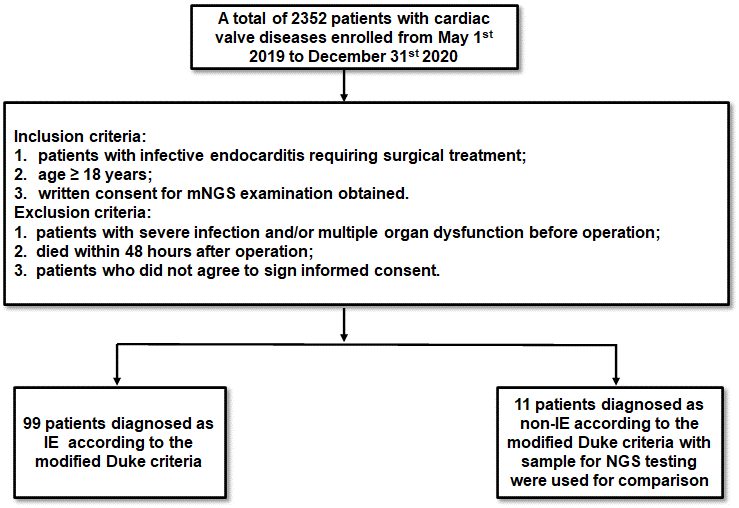


**Figure S2. ROC curves for different test modalities in patients with IE.**


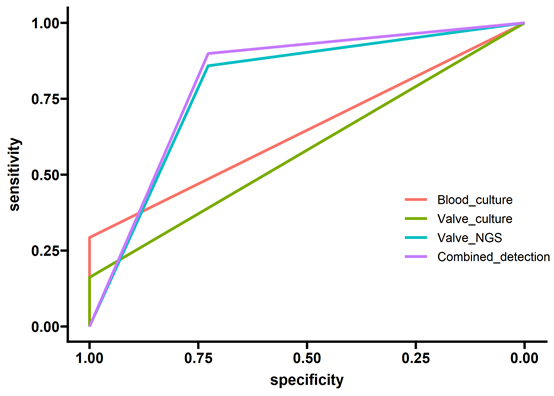

Supplement: Supplementary file 1 [file Data_Sheet_1.docx]
